# Commentary: Everybody hurts, sometimes: ERAS against opioids

**DOI:** 10.1016/j.xjon.2020.12.009

**Published:** 2020-12-23

**Authors:** Jenalee N. Coster, Bryan M. Burt

**Affiliations:** Division of Thoracic Surgery, Michael E. DeBakey Department of Surgery, Baylor College of Medicine, Houston, Tex

**Figure:**
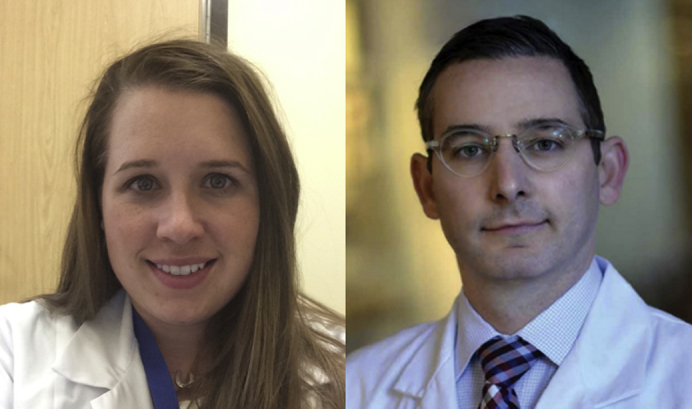
Jenalee N. Coster, MD, and Bryan M. Burt, MD

**Central Message**

Patients undergoing thoracic surgery are at high risk for opioid dependence, which may be mitigated by enhanced recovery protocols.

See Article page 173.

Opioid addiction is a major public health hazard in the United States, where prescription opioid overdoses were responsible for 41 deaths per day in 2018. Over the last several years, awareness of opioid addition and resolute changes in provider practices have decreased prescription opioid-related mortality by up to 13.5%^1^ and have empowered surgeons to address the opioid epidemic head-on. Enhanced recovery after surgery (ERAS) pathways that standardize perioperative pain medication regimens are likely an effective, understudied weapon against the overprescription of narcotics.

Patients undergoing thoracic surgery (open and minimally invasive) are at high risk for chronic pain and persistent opioid use,^2^ and modifiable risk factors contributing to these problems are not well understood. In this issue of *JTCVS Open*, Hodges and colleagues^3^ present their results of a preemptive analgesic ERAS protocol for patients undergoing foregut surgery (40%) or lung resection surgery (35%) to specifically evaluate factors associated with postoperative opioid use. Importantly, this protocol began with a preoperative educational component, an ambulation regimen, and cessation of smoking and alcohol (4 and 2 weeks before surgery, respectively). Their pain control regimen included tramadol and gabapentin in the immediate perioperative period, intraoperative intercostal nerve blocks with liposomal bupivacaine, and intraoperative intravenous acetaminophen and ketorolac. Postoperatively, patients were maintained on a scheduled regimen of acetaminophen, ketorolac, gabapentin, methocarbamol, and a Lidoderm patch. At the time of discharge, 72% of patients did not require opioids, and at the time of outpatient follow up, excellent patient-reported measures of pain control were observed.

Eight percent of patients in this study had a clinical diagnosis of opioid dependence before surgery, and 33 patients (15%) were taking prescribed opioids before their operation, showcasing the impact of the opioid epidemic on our population of thoracic surgical patients. Not surprisingly, preoperative opioid use was a significant risk factor for postoperative opioid use. In the majority of patients (85.5%) who were opioid-naïve before surgery, the authors identified tobacco use, American Society of Anesthesiologists classification 3 or 4, and any pain medication use as significantly associated with discharge opioid prescriptions. These are important data for informing patient and provider expectations of surgery and for potential tailoring or refinement of future ERAS protocols.

The astute reader, however, may wonder about the impact of chest surgery on these findings reported from cohorts of patients that also included a significant fraction of laparoscopic operations. In their comparison of abdominal and thoracic surgical procedures, Soneji and colleagues^4^ identified lung resection surgery as the strongest risk factor for postoperative opioid prescription in opioid-naïve patients. Practicing clinicians and surgeons know that patients with preoperative opioid dependence are challenging on many levels. After reading this article, one might surmise that this population could specifically benefit from an ERAS pathway, and we look forward to targeted studies in these patients. Taken together, the findings reported by Hodges and colleagues are significant and could contribute to further reductions in opioid prescriptions. We hope that the readers will appreciate the impact of ERAS protocols in thoracic surgery, formalize their expectations of recovery, and arm themselves to address the opioid epidemic in their own practices.
